# Hopelessness and Post-Traumatic Stress Symptoms among Healthcare Workers during the COVID-19 Pandemic: Any Role for Mediating Variables?

**DOI:** 10.3390/ijerph18126579

**Published:** 2021-06-18

**Authors:** Andrea Aguglia, Andrea Amerio, Alessandra Costanza, Nicolò Parodi, Francesco Copello, Gianluca Serafini, Mario Amore

**Affiliations:** 1 Department of Neuroscience, Rehabilitation, Ophthalmology, Genetics, Maternal and Child Health, Section of Psychiatry, University of Genoa, Largo Paolo Daneo, 3, 16132 Genoa, Italy; andrea.amerio@unige.it (A.A.); parodinicol@yahoo.com (N.P.); gianluca.serafini@unige.it (G.S.); mario.amore@unige.it (M.A.); 2 IRCCS Ospedale Policlinico San Martino, Largo Rosanna Benzi, 10, 16132 Genoa, Italy; francesco.copello@hsanmartino.it; 3 Department of Psychiatry, Faculty of Medicine, University of Geneva (UNIGE), 1206 Geneva, Switzerland; alessandra.costanza@unige.ch

**Keywords:** mental health, hopelessness, healthcare workers, post-traumatic symptoms, psychological trauma, anxiety, mental health promotion

## Abstract

The Coronavirus-19 (COVID-19) pandemic has many psychological consequences for the population, ranging from anxious-depressive symptoms and insomnia to complex post-traumatic syndromes. This study aimed to evaluate the impact of the Covid-19 pandemic on the mental well-being of healthcare workers, focusing on the association between hopelessness, death anxiety, and post-traumatic symptomatology. Eight hundred forty-two healthcare workers were recruited between 21 March 2020 and 15 May 2020. A specific questionnaire was administered to assess socio-demographic and clinical characteristics, together with psychometric scales: Beck Hopelessness Scale, Death Anxiety Scale (DAS), and Davidson Trauma Scale (DTS). Respondents with hopelessness scored higher in the DAS and DTS than respondents without hopelessness. Furthermore, death anxiety was identified as a potential mediator of the significant association between hopelessness and post-traumatic symptomatology. The impact of death anxiety should be recognized in vulnerable populations, such as frontline healthcare workers. Therefore, pharmacological and non-pharmacological strategies could be useful to attenuate the negative psychological consequences and reduce the burden worldwide.

## 1. Introduction

On 30 January 2020, Coronavirus-19 (COVID-19) disease was declared a public health emergency of international concern by the World Health Organization (WHO). On March 11th, the WHO Director General defined the occurrence of Covid-19 infection as a pandemic. Therefore, the governments of each nation hypothesized the negative socio-economic impact of the pandemic with detrimental consequences on local and worldwide health services [1]. For this reason, most governments obliged the general population to several security measures, including quarantine, lockdown, and confinement [2,3].

The combination of rapid spread and increased mortality rate of the pandemic has also provoked public health issues worldwide. Furthermore, the persistent fear against the new invisible enemy led to a significant increase of consequences on mental health in the general population [4,5,6,7,8,9], healthcare workers [7,9,10,11,12,13,14,15], and general practitioners [16]. Therefore, preventive strategies to limit the disease transmission and protect high-risk and vulnerable individuals were implemented [17].

Healthcare workers had to deal with never-before-seen stressful clinical conditions with a very high risk of adverse psychological outcomes. Several risk and protective factors may contribute to these outcomes, including individual (e.g., age, gender, and working status), interpersonal (e.g., marital status, having children, social and familial support), institutional (e.g., presence of protective equipment and rest guarantee), and community (e.g., stigma, anger or compassion of relatives of dead patients, social media). Furthermore, healthcare workers also had to deal with increased workload and physical exhaustion for the worsened clinical conditions of the infected patients, witnessing a higher-than-usual death rate associated with fear of contagion or death for themselves and colleagues [18]. Notably, frontline healthcare workers were most likely to experience psychological distress and psychiatric symptomatology, particularly post-traumatic stress symptoms [13,19,20,21,22].

Post-traumatic stress disorder (PTSD) occurs when someone has experienced or witnessed a life-threatening traumatic event, contributing to a substantial burden on individuals and society. Exposure to traumatic events is essential in diagnosing related symptoms. Conversely to common stressful events (e.g., natural disasters), the traumatic COVID-19 pandemic experience is represented by a pervasive, invisible, and intangible stimulus, determining a persistent crisis in exposed individuals. The real traumatic impact of the COVID-19 pandemic is quite unknown as it has been investigated only in terms of acute post-traumatic stress manifestations [23]. Several systematic reviews and clinical studies have evaluated post-traumatic stress symptoms in frontline healthcare workers, investigating clinical predictors, risk factors, and psychiatric manifestations [11,14,24,25,26,27,28,29,30,31,32]. However, to our knowledge, no studies have reported a potential mediator between hopelessness and post-traumatic stress symptoms. Beck et al. defined hopelessness as a negative view of the self and of the self in relation to the world and a future characterized by the lack of finding a solution for one’s problems [33]. Studies conducted in palliative care settings found that these feelings can be experienced by healthcare workers with the development of personal fear of death first, and later post-traumatic stress symptoms [34,35]. In the context of the Covid-19 pandemic, in light of many losses and ultimately the same feelings analogous to those that delineate the hopelessness concept [36], we hypothesized in this exploratory study that precisely the latter could act as a mediator with post-traumatic stress symptoms.

Therefore, this study aimed to analyze the impact of the COVID-19 pandemic on the mental well-being of healthcare workers in a large Italian hospital, with a focus on specific socio-demographic and clinical characteristics that may be more related to hopelessness as well as to the potential association with death anxiety and post-traumatic stress symptoms.

## 2. Materials and Methods

### 2.1. Sample

About 2500 healthcare workers of an Italian hospital were invited to submit a semi-structured online questionnaire between 21 March 2020 and 15 May 2020. The questionnaire was sent by email anonymously. The survey was conducted through the Google Forms online platform, keeping the individual identity and processing of personal data and the collected information confidential. Written informed consent explaining the nature and purpose of our research in detail was digitally signed by the individuals before participation in this study, agreeing to the analysis and use of their responses for research aims. Participants were consecutively recruited without any monetary remuneration.

Two pre-screening questions were made. The first to avoid potential bias due to the Covid-19 infection: “have you ever been infected by Covid-19 virus? (yes/no); and second, to determine if a healthcare worker was exposed to the Covid-19 infection: “have you ever been exposed directly or indirectly to Covid infection? (yes/no)”. A total of 352 were excluded due to a positive response. As done before [17,37], the permission to conduct the study was granted by the Dean of the Department (DINOGMI) and the General Director of the IRCCS Ospedale Policlinico San Martino.

### 2.2. Survey

The first part of the questionnaire included several socio-demographic and working characteristics of healthcare workers: gender, current age, marital status, educational level, having children or family members at risk, hours of sleep pre- and post- COVID-19 period, having chronic medical diseases, working role (e.g., medical doctor, nurse, or trainee), medical working area (e.g., medicine, surgery, or services), type of contract (fixed-term or permanent), and time spent on the internet and social networks to collect information about the COVID-19 pandemic.

The second part of the questionnaire specifically investigated different clinical dimensions through the administration of specific psychometric tools. First, the Beck Hopelessness Scale (BHS) [38]: this self-administered psychometric tool is widely used for self-assessment of hopelessness and consists of 20 items with the possible answers of true or false. Three factors have been identified: feelings for the future, loss of motivation, and future expectations. Second, the Death Anxiety Scale (DAS): this self-administered psychometric tool, developed by Templer in 1970 and validated in the Italian language by Saggino and Kline [39], was used to assess death anxiety. It consists of 15 items with the possible answers of true or false (e.g., “I am very afraid of dying”, “I am afraid of dying painfully”, and “The sight of a corpse horrifies me”). A high score reflects a higher level of death anxiety. Lastly, the Davidson Trauma Scale (DTS) [40]: this self-administered psychometric tool was used to assess the severity and frequency of general PTSD symptoms. This trauma scale is not specific to post-traumatic stress symptoms related to COVID-19. Each item of the scale has a score from zero to four for both severity and frequency.

### 2.3. Statistical Analysis

Continuous variables were represented as mean and standard deviation (SD), while categorical variables were represented as frequency and percentage considering socio-demographic and working characteristics. The sample was divided into two subgroups: the first consisted of respondents reporting hopelessness according to a BHS score equal to or greater than nine, while the second subgroup was characterized by respondents without hopelessness according to a BHS score lower than nine. This cutoff score was used because of the predictive value of suicidal behaviors [41]. Before comparing the two subgroups, the normality of the data distribution was assessed using the Kolmogorov–Smirnov test. The Student’s *t*-test for independent samples and Pearson’s test (chi-square, χ^2^) were performed for continuous and categorical variables, respectively.

Finally, a mediation analysis was carried out between hopelessness (independent variable) and the presence of post-traumatic stress symptoms (dependent variable) via death anxiety (mediator). Linear regression analyses were conducted, and the Sobel test was used to examine if death anxiety significantly mediated the relationship between hopelessness and post-traumatic stress symptoms [42].

All statistical analyses were performed using the SPSS version 25.0 (IBM Corp., Armonk, NY, USA), and the value of statistical significance was set at *p* < 0.05 (two-tailed).

## 3. Results

Overall, eight hundred forty-two (N = 842) respondents completed the online questionnaire, with a 39% response rate. The average age of the total sample was 41.79 ± 12.51 years (minimum 24, maximum 68), the number of male respondents was 273 (32.4%), while the married respondents were 464 (55.1%). Overall, 70% of the respondents included had at least one chronic general medical condition (N = 597), 65.2% worked mainly in the medical field (N = 549), while the three sub-categories (medical doctor, nurse, and trainee) were equally represented. The socio-demographic and clinical characteristics of the total sample are summarized in Table 1.

The total sample was then divided into two subgroups according to the presence or absence of hopelessness as defined by a BHS score greater than or equal to nine. The two subgroups had no statistically significant differences in terms of socio-demographic and working status, as reported in Table 1.

Regarding the investigated symptom dimensions, respondents with hopelessness scored higher in the DAS and DTS than those without hopelessness (BHS < 9).

**Figure 1:**
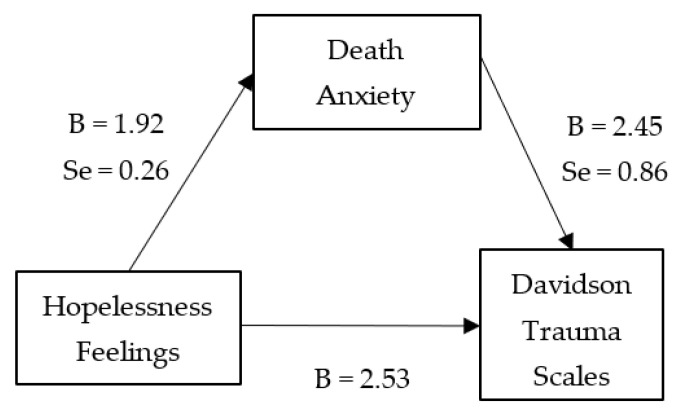
Mediation analysis between hopelessness and the development of post-traumatic symptoms.

From simple linear regression, hopelessness feeling was a statistically significant predictor of post-traumatic symptoms (B = 2.53, se = 0.79, t = 2.47, *p* = 0.04, 95% CI = 1.05–1.93). Furthermore, hopelessness feeling is also a significant predictor of the mediating variable (death anxiety, B = 1.92, se = 0.26, t = 7.44, *p* < 0.01, 95% CI = 1.41–2.42). Next, when the mediator, death anxiety, was entered in the regression analysis, hopelessness was no longer a significant predictor of post-traumatic stress symptoms. Finally, the Sobel test confirmed death anxiety as a potential mediator of the association between hopelessness and post-traumatic stress symptoms (Z = 2.66 and *p* = 0.007) (Figure 1).

## 4. Discussion

This study is, to our knowledge, the first to specifically investigate the impact of hopelessness among healthcare workers directly or indirectly exposed to patients with COVID-19 and the potential correlation between the presence of death anxiety and post-traumatic stress symptoms in this population.

The findings of our study show that the presence of hopelessness in healthcare workers who assisted and treated individuals with COVID-19 infection was associated significantly with higher mean scores on death anxiety and post-traumatic stress symptoms. Healthcare workers are considered a population vulnerable to a higher risk of psychological distress and psychiatric symptomatology and disturbances. From a recent review, the authors reported that between 11% and 73.4% of healthcare workers showed general psychopathology with different prevalence ranges: depressive symptoms in 27.5–50.7%, sleep disturbances in 34–36.1%, severe anxiety symptoms in 45%, and post-traumatic symptomatology during outbreaks, lasting after 1–3 years, in 10–40% of cases [21]. Furthermore, high stress levels with somatization related to working are reported in 18.1% to 80.1% [21]. Recent clinical studies confirmed these results, demonstrating a higher prevalence in young females with less working experience, particularly in nurses and in frontline staff compared to the medical doctor’ population and second-line staff, respectively [14,22,24,28,31,43].

No gender and working characteristic’ differences were found, contrary to current literature describing differences for frontline nurses in terms of well-being, feelings of burn-out, fear of self-infection, and of their performance and quality of care for the patients [14,24,32,44,45]. Due to the significant increase in working hours and number of COVID-19 patients who need to be assisted, the stressful clinical practice of frontline healthcare workers could have determined frustration, feelings of lower competence, and low-self-esteem directly related to the growing number of deaths during the current pandemic. Furthermore, job insecurity and inadequate personal equipment, long periods of isolation, uncertainty about the future, pre-existing psychological problems, an increase of perceived stress and threats, emotional and physical exhaustion, exposure to patient deaths, caregiver overload, and perceived degrees of threats associated with COVID-19 are considered as significant determinant factors of increased general psychopathology [11,14,25,27,29,30]. Lastly, persistent fear of infection and consequently an increased anxiety related to death, associated with other psychiatric clinical dimensions, as identified in existing studies [10,46], could occur in this population.

Based on the mediation analysis, death anxiety has been identified as a possible mediator of the association between hopelessness and post-traumatic stress symptoms. These results support the assumption that those with growing hopelessness may report clinically significant post-traumatic stress symptoms through the increased death anxiety.

In contrast to other common traumatic events (natural disasters, e.g., tsunami, earthquake, or civil war), which are generally localized to a specific area and a limited time and allow avoidance of the perceived danger related to trauma, the current pandemic constitutes a new form of traumatic stressful event in which the threat is not only difficult to localize but is also transferred from individuals to subjects invisibly, generating distrust, suspicion, and pervasive fear towards both unknown subjects and their closest relatives [19]. Furthermore, the loss of a loved one may be interpreted as one of the most intense and painful traumatic experiences that individuals may report. This is a very destabilizing event affecting the overall individual well-being in many ways; however, based on individual resilience and coping strategies, this traumatic experience may be appropriately processed in order to reach a functional recovery [47]. Furthermore, according to the imposed lockdown and restriction measures to limit the diffusion of the pandemic, the family members of subjects admitted in the hospital environment for COVID-19 infection were usually obliged to stay outside and had no access to medical wards, even for the last farewell before the patient died. This decision exposed healthcare workers to the traumatic experience of subjects who frequently died alone due to their severe clinical conditions but even to the need to act as an intermediary of the medical conditions to parents, with a consequent increase in emotional burden and consequent feelings of guilt and frustration [48,49,50]. The hopelessness of healthcare workers could be further aggravated by other safety and restriction measures adopted locally during the pandemic with a lower or absent possibility to practice physical, recreational, and social activities that are recognized as promoting physical and psychological well-being. Overall, the persistent hopelessness, generated by both the working burden and restriction measures of social distancing, associated with the increased deaths, may have contributed to post-traumatic clinical conditions among healthcare workers.

Therefore, it is crucial to pay greater attention to all social categories that may be defined as “at-risk”, such as healthcare workers, and promote locally targeted and specific prevention strategies such as implementing effective communication through an ad hoc helpline to provide adequate psychological support. These strategies may support an easier adaptation to lifestyle changes and facilitate the early diagnosis and adequate treatment of psychiatric symptoms. Furthermore, all health and preventive interventions should be integrated, providing a structured protocol to better approach COVID-19 patients with adequate personal protective equipment to reduce feelings of uncertainty, fear, and inadequacy.

From the perspective of clinical implications, several papers on how to help healthcare workers during the COVID-19 pandemic are emerging in the literature. These strategies are aimed at improving the mental health of healthcare workers, being focused on an integrated approach; intervention and mitigation measures address various levels, ranging from the individual to the organization of institutions [51,52]. The recommendations, subdivided into low, medium, and high resources requiring the corresponding degrees of mental health resources and infrastructures [51], concern practical interventions ranging from the organization of work and family-work balance to the clarification and standardization of procedures and decision-making, up to psychoeducational programs and psychotherapy, supportive included [52]. In a recent systematic review of the Cochrane Library [53], interventions to support the resilience and mental health of frontline healthcare workers during and after a disease outbreak, epidemic, or pandemic (including SARS, MERS, and COVID-19) were examined. The objectives of this work were two-fold: to assess the effects of interventions aimed at supporting resilience and to identify barriers and facilitators that can improve such interventions [53]. The authors highlight the current lack of both quantitative and qualitative evidence on the effects of such interventions. Similarly, barriers and facilitators remain uncertain. However, similarly to the studies mentioned above, they underline the need for an integrated model of action, focusing on both workplace characteristics and support of basic daily needs through to pharmacological and non-pharmacological interventions [52]. Other studies have instead addressed specific characteristics of exposed individuals (such as sensitivity to stigma, moral injury, some personality traits, the tendency to alexithymia, or a number of socio-ecological characteristics) [51,52,53,54], in order to characterize the vulnerability of these individuals and to identify more specific interventions. Overall, their findings are not conclusive. As asserted in the Cochrane Library systematic review, research in this field is current a real priority [53].

Importantly, the present study needs to be interpreted considering the following limitations/shortcomings: first, the small sample that was recruited only in a single university hospital and the low questionnaire response rate of 39% limit the generalization of our findings. Additionally, the cross-sectional design of the present study does not allow adequate information regarding the temporal correlation between the investigated variables. Moreover, no information is available on mental health status before the COVID-19 pandemic, which would be useful to better quantify the real impact of this pandemic on the recruited hospital’s healthcare workers. Particularly, because there is no baseline assessment of hopelessness or fear of death, we cannot have absolute certainty that these latter are anchored to COVID-19. However, in this context, a recently published paper relates death anxiety among social workers as a consequence of the COVID-19 pandemic [36]. Lastly, the PTSD symptoms have been investigated with a general evaluation scale, not specifically related to the COVID-19 pandemic.

In summary, it must be emphasized that these results need to be replicated in larger and more representative samples. This is in consideration of the two most limiting factors, represented by the low response rate and the preliminary cross-sectional design of the obtained data.

## 5. Conclusions

The psychological impact of death fear and anxiety, possibly induced by the rapid spread of the COVID-19 pandemic, should be recognized in vulnerable populations such as frontline healthcare workers. Pharmacological and non-pharmacological strategies could be useful for attenuating the occurrence of dramatic negative psychological consequences in healthcare workers and reducing the burden worldwide [55,56,57,58].

## Figures and Tables

**Table 1:** Socio-demographic and working characteristics of the total sample, and comparison of the two subgroups based on the presence of hopelessness.

	Total Sample (N = 842)	BHS ≥ 9 (N = 169)	BHS < 9 (N = 673)	t/X^2^	*p*
Gender (males), N (%)	273 (32.4)	61 (36.1)	212 (31.5)	1.30	0.254
Age, M ± SD	41.79 ± 12.51	43.38 ± 12.78	41.39 ± 12.42	−1.86	0.064
Marital Status, N (%)					
Single	296 (35.2)	46 (27.2)	250 (37.2)	6.35	0.096
Married	464 (55.1)	105 (62.1)	359 (53.3)		
Separated/divorced/widowed	82 (9.7)	18 (10.7)	64 (9.5)		
Educational level (years), M ± SD	17.75 ± 3.60	17.60 ± 3.83	17.79 ± 3.54	0.61	0.543
Children, N (%)					
No	475 (56.4)	88 (52.1)	387 (57.5)	2.52	0.283
Yes, they live with me	295 (35.0)	68 (40.2)	227 (33.7)		
Yes, they don’t live with me	72 (8.6)	13 (7.7)	59 (8.8)		
Family members at risk, N (%)	180 (21.4)	40 (23.7)	140 (20.8)	0.66	0.416
Hours of sleep, M ± SD					
Pre- COVID-19	7.04 ± 0.95	6.97 ± 1.01	7.05 ± 0.94	0.99	0.32
Post- COVID-19	6.45 ± 1.50	6.46 ± 1.52	6.45 ± 1.49	−0.12	0.903
Having Chronic Illness, N (%)	597 (70.9)	117 (69.2)	480 (71.3)	0.29	0.593
Working role, N (%)					
Medical Doctor	257 (30.5)	57 (33.7)	200 (29.7)	1.09	0.581
Nurse	257 (30.5)	48 (28.4)	209 (31.1)		
Trainee	328 (39.0)	64 (37.9)	264 (39.2)		
Medical working area, N (%)					
Medicine	549 (65.2)	121 (71.6)	428 (63.6)	4.04	0.133
Surgery	161 (19.1)	28 (16.6)	133 (19.8)		
Medical services	132 (15.7)	20 (11.8)	112 (16.6)		
Type of contract, N (%)					
Fixed-term contract	264 (31.4)	48 (28.4)	216 (32.1)	0.856	0.355
Permanent contract	578 (68.6)	121 (71.6)	457 (67.9)		
Searching for information, N (%)					
<1 h	202 (24.0)	47 (27.8)	155 (23.0)	3.25	0.354
1–3 h	498 (59.1)	100 (59.2)	398 (59.1)		
3–8 h	112 (13.3)	18 (10.7)	94 (14.0)		
>8 h	30 (3.6)	4 (2.4)	26 (3.9)		
DAS, M ± SD	7.59 ± 3.09	9.12 ± 3.09	7.21 ± 2.97	−7.44	<0.001 *
DTS, M ± SD	27.24 ± 15.68	29.26 ± 15.47	25.73 ± 15.71	−2.08	0.050 **

DAS = death anxiety scale; DTS = Davidson trauma scale; M = mean; SD = standard deviation. * Effect size Cohen’s d: 0.63; ** Effect size Cohen’s d: 0.23.

## Data Availability

The data are not publicly available due to privacy/ethical restrictions.
